# Species Richness, Abundance, and Vertical Distribution of Epiphytic Bromeliads in Primary Forest and Disturbed Forest

**DOI:** 10.3390/plants13192754

**Published:** 2024-09-30

**Authors:** Sugeidi S. Siaz Torres, Edilia de la Rosa-Manzano, Leonardo U. Arellano-Méndez, Karla M. Aguilar-Dorantes, José Guadalupe Martínez Ávalos, María Cruz Juárez Aragón

**Affiliations:** 1 Instituto de Ecología Aplicada, Universidad Autónoma de Tamaulipas, Avenida División del Golfo Núm, 356, Colonia Libertad, Ciudad Victoria 87019, Mexico; sugey_9876@hotmail.com (S.S.S.T.); luarellano@uat.edu.mx (L.U.A.-M.); jmartin@uat.edu.mx (J.G.M.Á.); mcjuarez@uat.edu.mx (M.C.J.A.); 2 Facultad de Ingeniería y Ciencias, Universidad Autónoma de Tamaulipas, Ciudad Victoria 87149, Mexico; 3 Centro de Investigación en Biodiversidad y Conservación, Universidad Autónoma del Estado de Morelos, Cuernavaca 62209, Mexico; karla.aguilar@uaem.mx

**Keywords:** gallery forest, Johansson zone, plant size, submontane scrub, *Tillandsia*

## Abstract

Epiphytes represent a key component in tropical forests. They are affected by anthropogenic and natural disturbances suffered by forests, since they depend on their hosts and the microclimatic conditions they generate. We analyzed differences in abundance, species richness, and vertical distributions of epiphytic bromeliads in primary and disturbed forests. We found a higher abundance (5316 individuals) and species richness (8 species) of bromeliads in disturbed forest than in primary forest (1360 individuals and 4 species, respectively). Most bromeliads (97%) were found on *Taxodium mucronatum*, a dominant tree with rough bark in the disturbed forest (gallery forest). Bromeliads were more abundant in the middle of the tree and diminished towards the trunk base and the upper crown. *Tillandsia baileyi* was the most abundant bromeliad, and the size categories of this species differentially colonize trees in gallery forest according to Johansson zones; seedlings of *T*. *baileyi* abundantly colonize the upper canopy, and juveniles colonize the middle canopy or secondary branches. Gallery forest represents an important reservoir for epiphytic bromeliads. Hence, it is important to extend this kind of study to wetland sites to understand the role they play as a habitat for epiphytes, as well as the dynamics and ecological processes that occur in such habitats.

## 1. Introduction

Tropical forests face serious problems due to land-use changes affecting biodiversity. Several studies have demonstrated that the number of epiphyte species is greater in conserved sites or tropical primary forest due to microclimatic conditions such as temperature, light, and humidity being more favorable for the establishment and growth of epiphytes, thereby helping to maintain their relationships with pollinators, dispersers, and mycorrhizas, among others [1,2,3]. Other studies have reported higher species richness and epiphyte abundance in isolated trees or secondary forests due to drought-tolerant species dominating modified ecosystems [4], while a few works found no differences [5,6]. Although vascular epiphytes are a major element of tropical forest ecosystems, our understanding of how disturbance affects epiphyte diversity is scarce. Epiphytes are considered an indicator of environmental quality, since they are sensible to microclimate shifts caused by anthropic disturbance [7]. In fact, the level of forest disturbance determines the plant composition and species richness.

Vascular epiphytes are relevant components of tropical forest biodiversity; they comprise over 31,000 species in 79 families and 918 genera, accounting for about 10% of total plants [8]. Epiphytes establish relationships with pollinators and dispersers in the canopy and form part of the water and nutrient cycle in tropical forests [9,10,11]. Among the best-represented families of vascular epiphytes are Orchidaceae, Araceae, Piperaceae, and Bromeliaceae [12,13]. The latter group is a Neotropical family integrated by a high diversity of terrestrial, saxicolous, and epiphytic species distributed from humid to arid environments [14]. In addition to their ecological contribution, bromeliads provide substantial ecosystem services in terms of capturing water, in addition to providing shelter and breeding sites for a variety of organisms, such as amphibians, bats, and mutualistic ants and spiders [10,15]; they are also a biological indicator of environmental disturbance and climate change [16].

A gallery forest or riparian forest is an ecosystem dominated by *Taxodium mucronatum* Ten. trees in the riparian corridors of major Mexican rivers [17,18]. This ecosystem provides landscape diversity and plays an important role in the ecotone dynamics of swamps and other wetlands [19,20]. Vascular epiphytes are rarely studied in gallery forests, maybe because such forests have lower abundance and species richness [21], in spite of individual trees such as *T. mucronatum* being abundant and dominant [22,23], which may increase habitat diversity for epiphytes, combined with the permanent presence of water. For example, members of the *Peperomia* genus have a marked preference for warm or temperate sites but are found in high-humidity environments such as gallery forest [24]. This ecosystem has been affected by agriculture and paddocks that have been established in surrounding areas, altering microclimatic conditions, including water flux [25], forming patches of vegetation on the borders of rivers or water channels, which affect the epiphyte community.

Other ecosystems, such as submontane scrub, are characterized by the prevalence of drought conditions for epiphytes due to low precipitation and high temperatures [26]. Species belonging to the *Tillandsia* genus are common in this ecosystem, since they possess morphological and anatomical traits such as narrow leaves, abundant trichomes, and crassulacean acid metabolism, avoiding water loss by transpiration [27,28]. Species such as *T. recurvata* L., *T. schiedeana* Steud., and *T. pringlei* S. Watson have also been reported in submontane scrub, although they are the least abundant species [29].

Vascular epiphytes show vertical distribution patterns on trees, indicating their ranges of tolerance to light, moisture, and other microclimatic factors [30]. Usually, epiphytes are more abundant in the intermediate zone on host trees and least abundant in the upper crown [10,31,32], where the prevalence of high radiation levels and less water availability are restrictive for some species [33,34]. The bark type of host trees plays an important role in successful dispersal, since rough bark is better at catching seeds than smooth bark, from which seeds can slip to the ground [35,36,37]. Additionally, host size is a determinant factor for the establishment of epiphytes, since taller individuals are older and have a greater probability of increasing epiphytic colonization [38].

The vertical distribution of vascular epiphytes on host trees can vary according to plant size. It has been suggested that adult (or larger) plants occupy the thickest branches because they can support more weight, while seedlings (or smaller plants) are distributed on the periphery or in the upper crown [39,40,41]. However, the vertical distribution of plant size in modified or disturbed ecosystems is poorly studied. Therefore, the success of epiphytes is related to the size and position they occupy in the host tree, among other factors. We propose the following questions: (a) How do the abundance, species richness, and vertical distribution of epiphyte bromeliads vary in forests with different degrees of conservation? (b) How does the vertical distribution of plant size of *Tillandsia baileyi* Rose ex Small differ in gallery forest? We hypothesize that (i) abundance and species richness are higher in gallery forest due to the frequency of high humidity, despite being a modified ecosystem, and (ii) the distribution of adult plants of *T. baileyi* is higher in the middle zone of host trees, while seedlings and juvenile plants occupy the upper and lower strata.

## 2. Results

### 2.1. Abundance, Species Richness, and Vertical Distribution of Bromeliads at Two Study Sites

**Figure 1:**
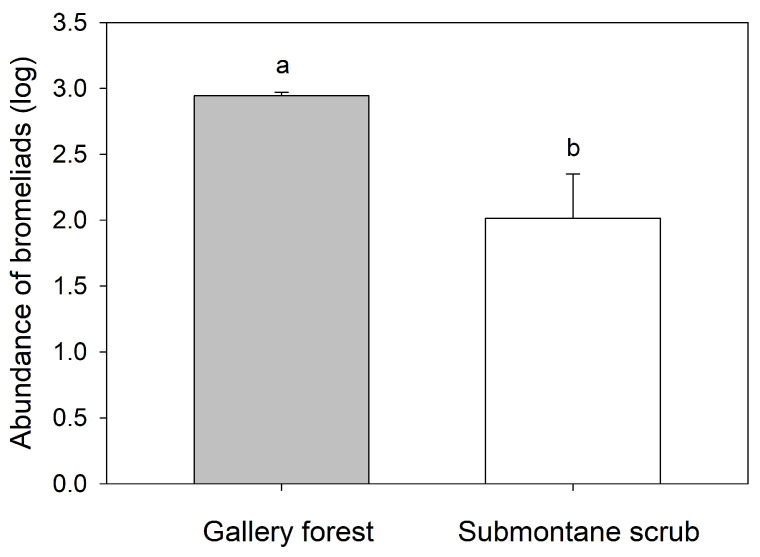
Abundance of epiphytic bromeliads in gallery forest and submontane scrub in Tamaulipas, Mexico. Each bar represents the mean ± SE. Different letters indicate significant differences with a *p* value of <0.05.

**Figure 2:**
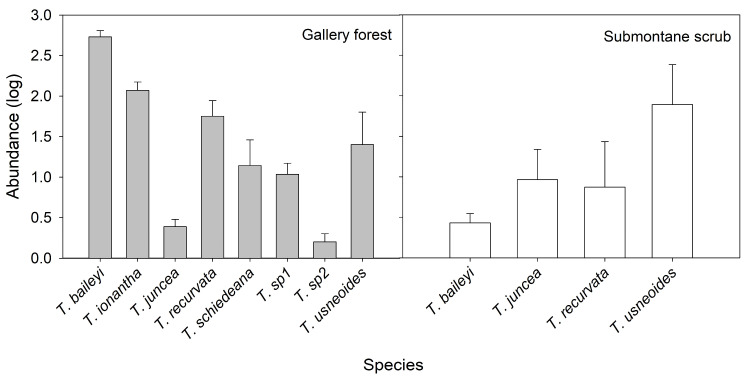
Abundance of Bromeliaceae species in gallery forest and submontane scrub in Tamaulipas, Mexico. Each bar represents the mean ± SE.

Species richness of bromeliads was two-fold in the gallery forest (eight species) in comparison to submontane scrub (four species). Additionally, the abundance of epiphytic bromeliads was higher in the gallery forest (5316 individuals) than in submontane scrub (1360 individuals) (*p* < 0.05; Figure 1). *Tillandsia baileyi* was the most abundant in the gallery forest (3467 individuals), followed by *T. ionantha* Planch. (811 individuals) and *T. usneoides* L. (688 individuals). In contrast, *T. usneoides* was the most abundant (1170 individuals) in submontane scrub, and *T. baileyi* was the least abundant (19 individuals) (Figure 2). In the gallery forest, *Taxodium mucronatum* had the highest relative abundance, coinciding with its high relative dominance and IVI (Table 1), while in submontane scrub, two host species (*Ehretia anacua* (Terán & Berland.) I. M. Johnst and *Ocotea tampicensis* (Meisn.) Hemsl.) had the highest relative abundance, relative dominance, and IVI. Despite *Quercus* sp. being fourth in IVI, it hosts half of the epiphyte bromeliads in submontane scrub (Table 2). We found one host species of each type of bark in the gallery forest, but in submontane scrub, 61.5% of the trees had smooth bark, followed by 30.8% with rugose bark and 7.6% with semi-rugose bark.

Study sites and Johansson zones influenced the abundance of epiphytic bromeliads (F_3,43_ = 15.1, *p* < 0.001). JZ2, 3, and 4 contributed positively to the model, while submontane scrub had a negative effect on the abundance of epiphytic bromeliads (Table 3). Differences were found only between JZ1 and other zones (JZ2, JZ3, and JZ4; *p* < 0.001; Table S1; Figure S2). Moreover, the abundance of epiphytic bromeliads differed between sites (*p* < 0.001).

### 2.2. Distribution of T. baileyi in Two Forests and on Host Tree Species

**Figure 3:**
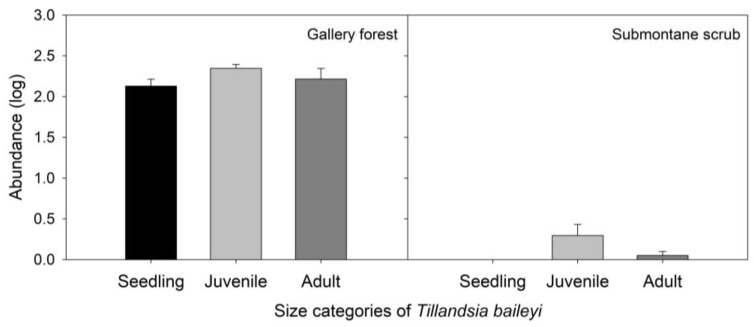
Abundance of *Tillandsia baileyi* based on size categories in gallery forest and submontane scrub in Tamaulipas, Mexico. Each bar represents the mean ± SE.

**Figure 4:**
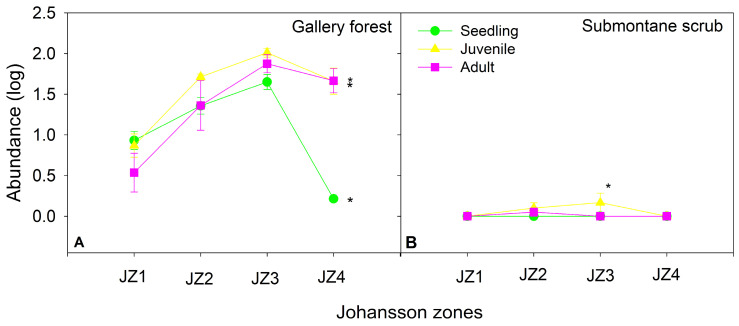
Size categories (seedling, juvenile, and adult) of *Tillandsia baileyi* based on Johansson zone in the gallery forest and submontane scrub in Tamaulipas, Mexico. Each point represents the mean ± SE; asterisk indicate significant differences among Johansson zones for each size category with *p* < 0.05.

The abundance of *T. baileyi* significatively differed among size categories (F_2,32_ = 3.37, *p* < 0.05) and between study sites (F_1,32_ = 270.15, *p* < 0.001) (Figure 3). Submontane scrub was the only variable that significantly contributed to the model, although it was negative (*p* < 0.001) (Table S2). The only size-pair category that was significant was juvenile vs. seedling *T. baileyi* (*p* < 0.05) (Table S3). The abundance of each size category of *T. baileyi* was significantly differed among JZs in the gallery forest (X^2^ ≤ 494.99, gl = 3, *p* < 0.001; Figure 4). However, in submontane scrub, only the juvenile category showed differences among the JZs (X^2^ ≤ 19.143, gl = 3, *p* < 0.005; Figure 4).

**Figure 5:**
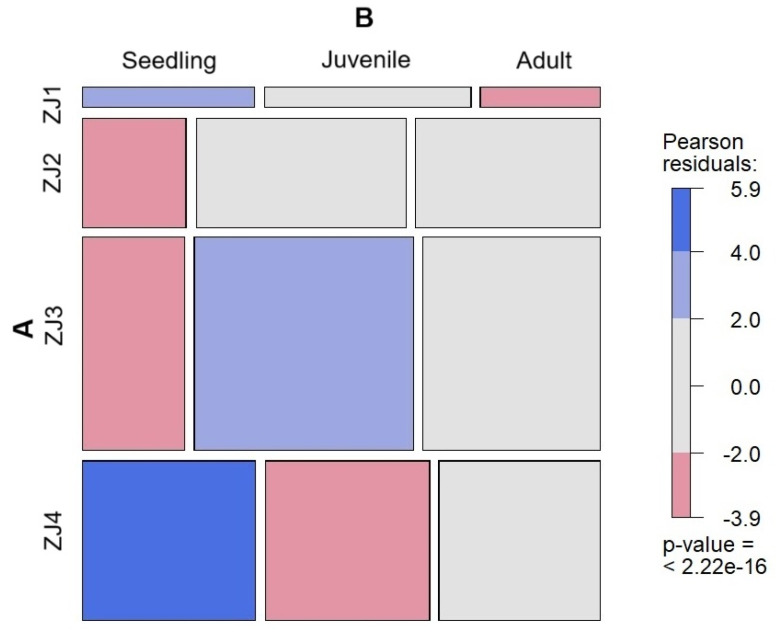
Mosaic display of the standardized residuals of (**A**) Johansson zones (JZ1, JZ2, JZ3, and JZ4) and (**B**) size categories of *Tillandsia baileyi* in the gallery forest. Blue boxes indicate a significant association between epiphyte species and hosts (*p* < 0.05). Association was considered positive when standardized residual values were >2 and negative when they were <−2 [42].

Contingency table analysis showed that the distribution of *T. baileyi* by category size is not homogeneous in the Johansson zones of host trees in the gallery forest (X^2^ = 87.5, df = 6, *p* ˂ 0.001, Table S4). Standard residual analysis indicated that seedlings of *T. baileyi* were significantly more abundant than expected by chance in Johansson zones 1 and 4, where seedlings have a 34% chance of occurrence. Moreover, juvenile plants of this species were more abundant in Johansson zone 3, with 43% of occurrence on trees in the same forest (Figure 5).

## 3. Discussion

Epiphytes are generally more abundant and species-rich in primary forests than in disturbed or modified landscapes, a pattern observed in diverse ecosystems [1,7,43,44,45,46]. For instance, 178 species of vascular epiphytes were recorded in primary forest, compared with only 81 species found in secondary forest in the Venezuelan Andes [43]. However, our results did not fulfill this expectation, as both the abundance and species richness of bromeliads were higher in the gallery forest than the disturbed forest. This unexpected result could be explained by the ability of certain epiphyte species to thrive in disturbed environments or on isolated trees due to their drought-adaptive traits [45,47,48,49]. For example, some ecosystems, such as wetlands, are characterized by high humidity and support greater epiphyte biodiversity, despite human disturbance being less pronounced in such environments [46,50,51].

Although the gallery forest represents a disturbed site, the species richness of bromeliads was comparable to that found in drier, more conserved habitats, such as the tropical dry forest and the submontane scrub in the Biosphere Reserve of “El Cielo” [29], where species richness is relatively lower. Despite its disturbance, the gallery forest supports a relatively high abundance of specific bromeliad species, while generally known for hosting only 8% of Mexico’s total epiphyte species (144 species) [21]. In our study, atmospheric bromeliads like *Tillandsia baileyi*, *T. ionantha*, and *T. usneoides* were most abundant in the gallery forest. Similarly, *T. usneoides* and *T. recurvata* were dominant in the submontane scrub. These species cataloged as atmospheric epiphytes have morphological adaptations such as narrow leaves and abundant trichomes, which enhance their ability to capture atmospheric water and nutrients, facilitating their survival in disturbed or drier environments [52]. In contrast, less abundant tank bromeliads such as *T*. sp1 and *T*. sp2 exhibit adaptations like broad, flat leaves that form water-holding chambers, which allow them to capture and store water from fog or dew. This water retention capability likely increases resilience in the species in drier and sun-exposed environments [53]. These ecological adaptations offer a potential explanation for the success of bromeliads in both disturbed and conserved sites, highlighting the complex relationship between disturbance and epiphyte diversity.

On the other hand, low abundance and species richness in the submontane scrub coincides with previous findings with respect to the same vegetation type within the “El Cielo” Biosphere Reserve, where epiphytes were also found to be less abundant than semideciduous and tropical montane cloud forest [29]. This may be attributed to the environmental conditions in submontane scrub characterized by high irradiance and drought [26], where trees and shrubs have broad crowns that expose epiphytes to increased light levels. Only species adapted to these stressful conditions, such as drought-tolerant bromeliads, can survive under the intense radiation in this habitat. For instance, *T. usneoides,* one of the few species present, has small leaves (43 mm leaf length; [52]) covered by abundant trichomes that aid in water absorption and uses crassulacean acid metabolism (CAM) to minimize water loss through transpiration [52]. Another plausible factor contributing to low epiphyte abundance is the prevalence of trees with smooth bark; in submontane scrub, approximately 70% of trees have smooth bark, which lacks the surface irregularities necessary to anchor fragile coma hairs.

In contrast *T. baileyi*, an atmospheric epiphyte, was highly abundant in the gallery forest, constituting 97% of all bromeliads and colonizing 89% of *T. mucronatum* trees, which were the primary host in this ecosystem (Table 1). While the abundance of *T. baileyi* was notable in our study, its overall distribution in Mexico has been declining. Previously recorded across southern Mexico [54], recent reports suggest its current distribution is limited to the northeast [21], likely due to the extensive transformation of tropical dry forest caused by agricultural expansion and human disturbance [55]. The tropical dry forest, once a suitable habitat for *T*. *baileyi,* [54], may no longer support its populations effectively.

Our study indicates that *T. mucronatum* trees, which grow in disturbed gallery forest areas surrounded by agricultural land, play a critical role in supporting epiphytic bromeliads. These trees are large and old and have extensive, well-branched canopies (the widest tree has a DBH of 228 cm)—traits that enhance their suitability as phorophytes. Additionally, their crowns provide a heterogeneous microenvironment for epiphytes, remaining leafy for most of the year, except from December to January, when sunlight is lowest [56]. This structural complexity is crucial for bromeliad colonization, as tree height and branch circumference have been positively correlated with the abundance of epiphytic bromeliads in different forest types, including the Atlantic Rainforest [57] and tropical forests [58]. Larger trees offer multiple shaded habitats, which are favorable for the growth of epiphytes [40,59,60].

Moreover, *T. mucronatum* may function as remanent tree species in gallery forest, providing essential support structures for epiphytic bromeliads. Studies have shown that isolated trees can harbor a significant number of epiphyte species; for example, one tree was found to support 34 epiphytic species, compared to a maximum of 66 species in primary forest [43]. This underscores the importance of remanent hosts for the conservation of epiphytes. It is also unlikely that seed dispersal limitations explain the abundance patterns observed in the gallery forest, as epiphyte seeds have been shown to disperse over distances of up to 2 km from their source [61]. Despite being surrounding by agricultural land, isolated patches of tropical dry deciduous forest may serve as a viable seed source for epiphytic colonization in the gallery forest.

Additionally, cypress (*Taxodium* sp.) host trees exhibit rough bark that facilitates the anchoring of bromeliad seeds, as similarly observed with *Taxodium ascendens* Brongn in Florida, USA; this rough bark hosts abundant *Tillandsia circinatta* Schlecht [62]. The rough bark of these trees presents fissures and cavities that can accumulate dust, moisture, and nutrients, providing an advantageous microenvironment for epiphytes [35,63]. In this study, cypresses growing along creek edges were found to benefit from the permanent availability of water, which contributes to a humid microclimate that mitigates the harsh conditions typically found in epiphytic habitats. This is analogous to findings in a tropical dry forest, where bromeliad abundance was found to decrease with distance from cenotes (water holes), as sites closer to cenotes experience higher nighttime humidity, which contributes to the maintenance of the bromeliad community [64].

Our results indicate that epiphytic bromeliads were more abundant in Johansson zones 2, 3, and 4 at both study sites, where host trees exhibit well-branched canopies. This pattern aligns with other studies suggesting that intermediate zones (JZ2 and 3) on the host trees provide more colonization area, favorable microclimates, and increased survival chances for epiphytes [10,31,34,65].

The abundance of *T. baileyi* sizes varied significantly between seedlings and juveniles, with fewer seedlings indicating low recruitment rates. While other clonal species also showed low seedling recruitment despite high-speed production [66,67,68], it is generally observed that smaller plants are more susceptible to desiccation during the dry season, which is a critical phase for epiphyte establishment. Seedlings are particularly vulnerable in the epiphytic habitat, which poses a significant challenge for their survival [68]. Understanding population structure is crucial when implementing conservation strategies, especially when detailed demographic data are lacking [69].

*Tillandsia baileyi* displayed differential colonization patterns on *T. mucronatum* host trees, preferring juvenile and adult individuals in the middle canopy (JZ3), while seedlings were more frequently found in the upper canopy or twigs (JZ 4) (Figure 5, Table S4). The higher-than-expected number of seedlings in the upper canopy of the gallery forest suggests that the dense foliage of *T. mucronatum* may mitigate harsh microclimatic conditions, such as intense solar radiation and low humidity [70], thereby facilitating colonization in these zones. This is similar to *Tillandsia schiedeana* in tropical dry forests, which colonizes the outer crown of *Bursera simaruba* (L.) Sarg., showing drought adaptations throughout its morphophysiology [37]. The preference of juvenile *T. baileyi* for the middle canopy (JZ3), which offers more stable microhabitats, further supports the notion that well-structured host trees are essential for epiphytic colonization [10].

## 4. Materials and Methods

### 4.1. Study Site

This study was carried out at two sites in Tamaulipas. (1) The first site was ejido La Cabecera in the municipality of Aldama, which is a gallery forest with anthropic disturbance, which was considered a disturbed forest. Gallery forest comprises groups of trees that develop along more or less permanent water courses. The vegetation in gallery forests is heterogeneous, with tree species that can reach 4 to 40 m in height, including numerous climbers and epiphytes. Some of the representative genera are *Salix*, *Taxodium*, *Acer*, *Inga*, *Carya*, *Fraxinus*, and *Alnus* [17]. (2) The second site was ejido Carricitos in the municipality of San Nicolas, which is characterized by generally unarmed, 3 to 5 m high and dense submontane scrub; this site was called primary forest. While dominant tree species vary from one region to another, the most frequent are *Helietta parvifolia* (A. Gray ex Hemsl.) Benth, *Neopringlea integrifolia* (Hemsl.) S. Watson, *Gochnatia hypoleuca* (DC.) A. Gray, *Pithecellobium brevifolium* Benth, *Quercus fursiformis* Small, and *Cordia boissieri* A.DC [17]. At both sites, a semi-warm, sub-humid climate predominates, with summer rains, presenting an average annual temperature higher than 18 °C and annual rainfall ranging from 900 to 1000 mm (UNAFOR 2803 and 2801, 2010).

### 4.2. Data Collection

Six 50 × 5 m linear transects were established at each study site (1.5 ha per site). In each transect, the tree species were identified. Subsequently, each tree was measured for diameter at breast height (DBH) and height using a tape measure and a distance meter (D210; Leica, Wetzlar, Germany), respectively. Only trees with a DBH greater than 10 cm were considered, since they offer a great diversity of ecological niches due to their variety of sizes and positions of their branches, facilitating colonization by epiphytes [71]. Identification of the trees at the species level was carried out using taxonomic keys and with the help of a specialist from the Institute of Applied Ecology of the Autonomous University of Tamaulipas. Tree bark was classified into the following three categories [72]: rugose (with deep grooves and ridges), semi-rugose (with small grooves and ridges), and smooth (lacking peeling or cracks).

Individuals of all epiphytic bromeliads at both study sites were recorded by direct observation with the aid of binoculars (EO-D102; Eagle optics, Middleton, WI,, USA). When the epiphytic individuals were at a distance greater than 5 m from the ground, a simple rope technique [73] was used to ascend to the canopy. The height of the bromeliads on the host was measured using a distance meter (D210, Leica, Wetzlar, Germany). Clones from rhizomatous plants or clumps of plants were considered one individual.

Since *T. baileyi* was the most abundant species in the gallery forest, the size categories of this species were analyzed to determine its structure and vertical distribution. *T. baileyi* individuals were classified into the following three categories according to size: seedling, juvenile, and adult. The seedling individuals measured between 1 and 10 cm in height without inflorescence, juveniles were between 11 and 25 cm tall and could present inflorescence or not, and adult plants measured more than 26 cm in height and presented inflorescence (Figure S1). Plant height was considered from the base to the longest leaf of the plant (Figure S1).

Vertical distribution of epiphytic bromeliads in the host was established through the following categories proposed in [63] and modified in this work: Zone 1 (JZ 1) corresponds to the total portion of the trunk, Zone 2 (JZ 2) is the lower canopy or first branches, Zone 3 (JZ 3) concerns the middle canopy of secondary branches, and Zone 4 (JZ 4) represents the upper canopy or twigs.

### 4.3. Data Analysis

Data were analyzed with R software version 4.0.4 [74]. The relative abundance of species was calculated as the number of individuals of each species/total abundance × 100. We assessed the importance value index (IVI) to ascertain the dominant tree species in each forest [75]. For details of IVI calculation, see [37]. If the IVI value for different species is similar, they contribute similarly to the composition, structure, site quality, and dynamics of the forest [76]. A one-way ANOVA was used to analyze the differences in abundance between study sites. 

The effects of the Johansson zones (JZ1, JZ2, JZ3, and JZ4) and sites on the abundance of epiphytic bromeliads and the effects of Johansson zones and the size categories (seedling, juvenile, and adult) of *T. baileyi* on the abundance of *T. baileyi* were evaluated using generalized linear models. To correct overdispersion, a quasi-Poisson distribution and a logit link function were used [77]. An analysis of variance (ANOVA) was applied to determine the significance of the following factors: Johansson zone, size category of *T. baileyi,* and site [78]. Normality was checked using the Shapiro–Wilk test. Subsequently, multiple means comparisons (Holm, *p* ˂ 0.05) were performed to evaluate possible differences between sites, Johansson zones, and size categories for *T. baileyi*. Holm’s method is a *p*-value adjustment used in multiple-hypothesis testing to avoid false statistical inferences [79]. An χ^2^ analysis was performed to evaluate the possible significant differences among the Johansson zones for each studied species. A contingency table with three size categories (rows) and four Johansson zones (columns) was structured. The association of size categories with Johansson zones of the gallery forest was evaluated with an χ^2^ test [80]. The frequency of the expected abundance of bromeliads was obtained with the product of the row and column totals divided by the total number of observations. When the χ2 test was significant (*p* ≤ 0.05), a standardized residual analysis was performed [42]. The association between the size categories and Johansson zones, on the one hand, was considered positive when the observed values were higher than the expected values and the values of the standardized residuals were >2. A negative association was considered when the observed values were lower than the expected values and standardized residual values were <−2 [42].

## 5. Conclusions

The gallery forest, despite being surrounded by agricultural land, is an important reservoir for epiphytic bromeliads. This means that large and old trees of *T. mucronatum* offer a great microsite to epiphytic bromeliads are crucial for their conservation and the maintenance of the species richness of the site. *T. baileyi* was the most abundant species in the gallery forest, and seedling was the least abundant category size, abundantly colonizing the upper crown, maybe in response to the dry and bright conditions prevalent in the outer crown. Our results represent a tool to propose conservation initiatives and management programs in the gallery forest, especially for *T. baileyi*, a species that has reduced its distribution in recent years [21]. According to NatureServe, *T. baileyi* is considered an endangered species in the USA [81], but in Mexico, its ecological and physiological requirements are unknown. Therefore, this study establishes a baseline for the conservation of this species. Research in gallery forest is essential and should be extended to wetland sites to better understand the role it plays as a habitat for epiphytes and the dynamics and ecological processes that occur in such habitats.

## Figures and Tables

**Table 1:** Host-tree traits from the gallery forest (DBH ≥ 10 cm), including bark type, relative abundance (%), relative frequency, relative dominance (%), importance value index (IVI), and epiphyte abundance (%).

Tree Species	Family	Bark Type	Relative Abundance (%)	Relative Frequency (%)	Relative Dominance (%)	IVI 100%	Epiphyte Abundance (%)
*Taxodium mucronatum*	Cupressaceae	Rugose	88.88	60	80.08	76.34	97.31
*Ficus* sp.	Moraceae	Smooth	8.35	30	19.89	19.40	2.60
*Tabernaemontana alba* Mill.	Apocynaceae	Semi-rugose	2.77	10	0.03	4.26	0.09
Total			100	100	100	100	100

**Table 2:** Host-tree traits in submontane scrub (DBH ≥ 10 cm), including bark type, relative abundance (%), relative frequency, relative dominance (%), importance value index (IVI), and epiphyte abundance (%).

Tree Species	Family	Bark Type	Relative Abundance (%)	Relative Frequency (%)	Relative Dominance (%)	IVI 100%	Epiphyte Abundance (%)
*Ehretia anacua*	Boraginaceae	Rugose	20.00	12.90	30.61	21.17	25.12
*Ocotea tampicensis*	Lauraceae	Smooth	24.71	12.90	15.63	17.75	2.29
*Zanthophyllum fagara* (L.) Sarg.	Rutaceae	Rugose	11.76	9.68	8.97	10.14	7.74
*Quercus* sp.	Fagaceae	Rugose	5.88	9.68	14.78	10.11	49.85
*Havardia pallens* (Benth.) Britton & Rose.	Fabaceae	Smooth	8.24	6.45	4.99	6.56	2.07
*Sebastiana pavonia* Muell.	Euohorbiaceae	Semi-rugose	7.06	9.68	2.39	6.37	1.69
*Pithecellobium flexicaule* (Benth.) Coult.	Fabaceae	Rugose	5.88	6.45	5.94	6.09	0.61
*Casimiroa greggi* (S.Watson) F.Chiang	Rutaceae	Smooth	3.53	9.68	4.68	5.96	0
*Ugnadia speciosa* Endl.	Sapindaceae	Smooth	3.53	6.45	3.09	4.36	7.73
*Randia obcordata* S. Watson	Rubiaceae	Smooth	3.53	3.23	5.30	4.02	0.15
*Acacia coulteri* Benth.	Fabaceae	Smooth	3.53	6.45	1.46	3.81	0
*Persea liebmannii* Mez	Lauraceae	Smooth	1.18	3.23	1.83	2.08	2.75
*Robinsonella discolor* Rose & Baker f. ex Rose	Malvaceae	Smooth	1.18	3.23	0.34	1.58	0
Total			100	100	100	100	100

**Table 3:** Generalized linear model with quasi-Poisson distribution where the variables of Johansson zone (JZ2, JZ3, and JZ4) and the submontane scrub were evaluated. Variable JZ1 and the gallery forest are not presented as coefficients because they were taken as reference points. *p* value < 0.001.

	Estimate	Standard Error	t Value	Pr(>|t|)
Intercept	3.730	0.394	9.45	0.001
JZ2	1.759	0.416	4.22	0.001
JZ3	2.116	0.411	5.14	0.001
JZ4	1.673	0.422	3.96	0.001
Submontane scrub	−1.324	0.197	−6.70	0.001

## Data Availability

In accordance with Open Science communication practices, the authors declare that all data are available within the manuscript.

## Supplementary Materials

The following supporting information can be downloaded at: https://www.mdpi.com/article/10.3390/plants13192754/s1, Figure S1. Size categories of bromeliad *T. baileyi*: (A) seedling; (B) juvenile; (C) adult. The diameter of the coin is 2.5 cm. Figure S2. Abundance of epiphytic bromeliads based on Johansson zone in gallery forest and submontane scrub in Tamaulipas, Mexico. Each bar represents the mean ± SE. Table S1. Paired comparisons between the Johansson zones of the gallery forest and submontane scrub in Tamaulipas, Mexico. JZ = Johansson zone. Table S2. Generalized linear model with quasi-Poisson distribution where the size categories of *Tillandsia baileyi* were evaluated. The adult category and the gallery forest were taken as reference points (*p* < 0.001). Table S3. Paired comparisons for size categories of *Tillandsia baileyi* in the gallery forest and the submontane scrub in Tamaulipas, Mexico. Table S4. Observed values and standardized residuals showing the association between size categories of *Tillandsia baileyi* and the Johansson zones of trees from the gallery forest. Association was considered positive when standardized residual values were >2, indicating that individuals of *T. baileyi* were more abundant than expected by chance and negative when residuals were <−2, suggesting that individuals of this bromeliad were less abundant than expected by chance [42].
