# Supplementary material for: Species Richness, Abundance, and Vertical Distribution of Epiphytic Bromeliads in Primary Forest and Disturbed Forest
Source: Plants (Basel). 2024 Sep 30;13(19):2754. doi: 10.3390/plants13192754 (PMC11478883; doi:10.3390/plants13192754)
Supplement: Supplementary file 1 [file plants-13-02754-s001.zip › Table S1 Plants.pdf]

Table S1. Paired comparisons between the Johansson zones of the gallery forest and the submontane scrub, Tamaulipas, Mexico. JZ = Johansson zone.

			**Estimate**	**z value**	**Pr(>|z|)**
JZ1	-	ZJ2	-1.759	-4.22	0.001
JZ1	-	ZJ3	-2.116	-5.14	0.001
JZ1	-	ZJ4	-1.674	-3.96	0.001
JZ2	-	ZJ3	-0.357	-1.87	0.121
JZ2	-	ZJ4	0.085	0.40	0.687
JZ3	-	ZJ4	0.442	2.18	0.086
