# Supplementary material for: Species Richness, Abundance, and Vertical Distribution of Epiphytic Bromeliads in Primary Forest and Disturbed Forest
Source: Plants (Basel). 2024 Sep 30;13(19):2754. doi: 10.3390/plants13192754 (PMC11478883; doi:10.3390/plants13192754)
Supplement: Supplementary file 1 [file plants-13-02754-s001.zip › Table S2 Plants.pdf]

Table S2. Generalized linear model with quasipoisson distribution where the size categories of *Tillandsia baileyi* were evaluated. The adult category and the gallery forest were taken as reference points. P < 0.001.

	**Estimate**	**Standard Error**	**t value**	**Pr(>|t|)**
**(Intercept)**	5.278	0.119	44.18	0.001
**Juvenile**	0.159	0.162	0.97	0.335
**Seedling**	-0.291	0.182	-1.59	0.12
**Submontane scrub**	-5.197	0.944	-5.50	0.001
