# Supplementary material for: Species Richness, Abundance, and Vertical Distribution of Epiphytic Bromeliads in Primary Forest and Disturbed Forest
Source: Plants (Basel). 2024 Sep 30;13(19):2754. doi: 10.3390/plants13192754 (PMC11478883; doi:10.3390/plants13192754)
Supplement: Supplementary file 1 [file plants-13-02754-s001.zip › Table S3 Plants.pdf]

Table S3. Paired comparisons for size categories of *Tillandsia baileyi* in the gallery forest and the submontane scrub, Tamaulipas, Mexico.

			**Estimated**	**Standard Error**	**Z value**	**Pr(>|z|)**
Adult	-	Juvenile	-0.159	0.162	-0.97	0.327
Adult	-	Seedling	0.291	0.182	1.59	0.220
Juvenile	-	Seedling	0.450	0.176	2.55	**0.032**
