# Supplementary material for: Species Richness, Abundance, and Vertical Distribution of Epiphytic Bromeliads in Primary Forest and Disturbed Forest
Source: Plants (Basel). 2024 Sep 30;13(19):2754. doi: 10.3390/plants13192754 (PMC11478883; doi:10.3390/plants13192754)
Supplement: Supplementary file 1 [file plants-13-02754-s001.zip › Table S4 Plants.pdf]

Table S4. Observed values and standardized residuals showing the association between size categories of *Tillandsia baileyi* and the Johansson zones of trees from the gallery forest. Association was considered as positive when standardized residual values were >2, indicating that individuals of *T. baileyi* were more abundant than expected by chance and negative when residuals were <–2, suggesting that individuals of this bromeliad were less abundant than expected by chance [50].

	**JZ1**		**JZ2**		**JZ3**		**JZ4**	
	observed	residuals	observed	residuals	observed	residuals	observed	residuals
**Seedling**	49	**2.460**	156	-3.390	298	-5.975	379	**8.294**
**Juvenile**	59	0.392	315	1.386	640	**4.118**	359	-5.767
**Adult**	34	-2.667	276	1.688	517	1.248	353	-1.681
