# Supplementary figures and images for: Species Richness, Abundance, and Vertical Distribution of Epiphytic Bromeliads in Primary Forest and Disturbed Forest
Source: Plants (Basel). 2024 Sep 30;13(19):2754. doi: 10.3390/plants13192754 (PMC11478883; doi:10.3390/plants13192754)

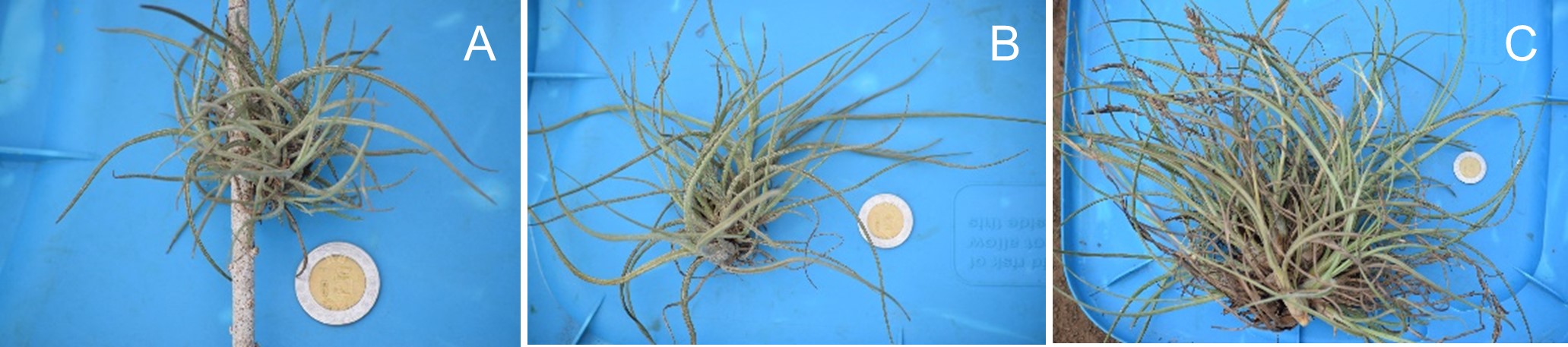

Supplement: Supplementary file 1 [file plants-13-02754-s001.zip › Figure S1.jpg]

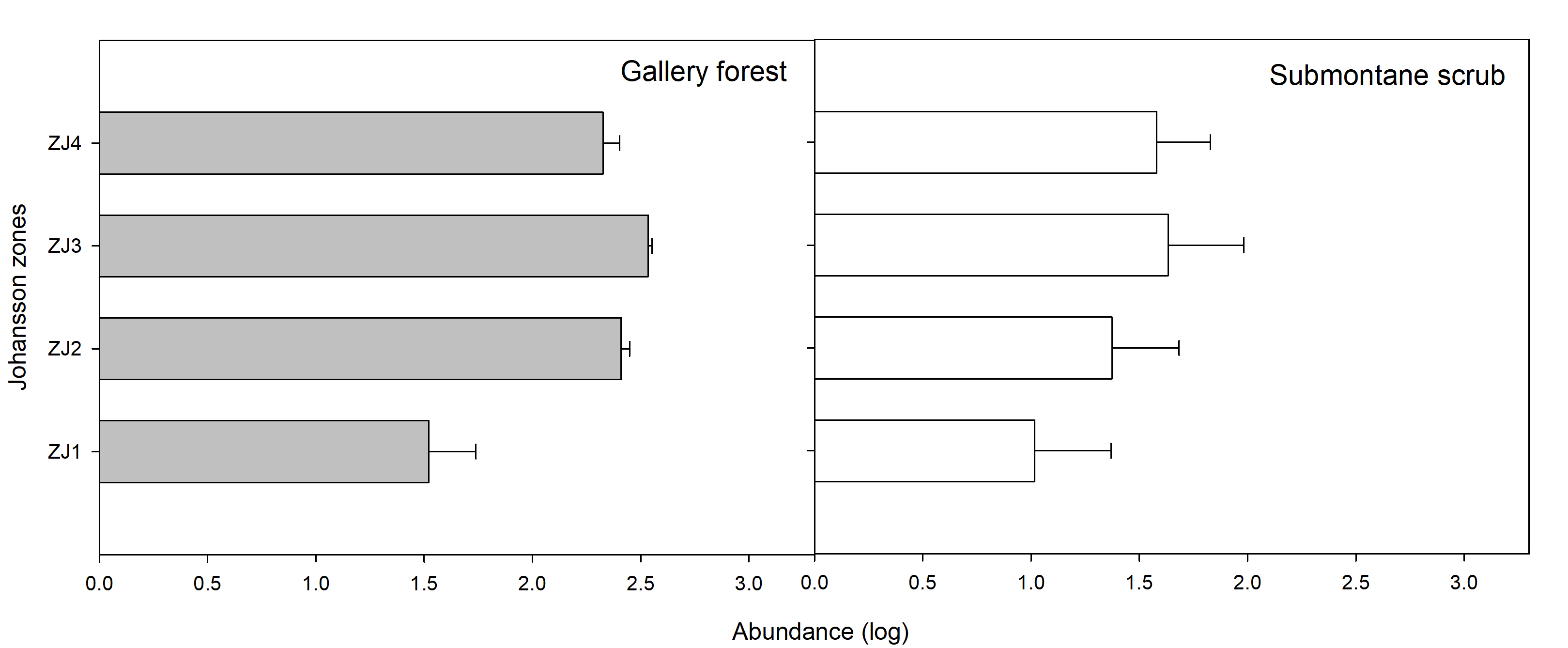

Supplement: Supplementary file 1 [file plants-13-02754-s001.zip › Figure S2.JPG]
